# Gene Coexpression Network Characterizing Microenvironmental Heterogeneity and Intercellular Communication in Pancreatic Ductal Adenocarcinoma: Implications of Prognostic Significance and Therapeutic Target

**DOI:** 10.3389/fonc.2022.840474

**Published:** 2022-06-01

**Authors:** Chengsi Wu, Yizhen Liu, Dianhui Wei, Li Tao, Lili Yuan, Tiantian Jing, Boshi Wang

**Affiliations:** ^1^ State Key Laboratory of Oncogenes and Related Genes, Shanghai Cancer Institute, Renji Hospital, Shanghai Jiao Tong University School of Medicine, Shanghai, China; ^2^ Department of Medical Oncology, Fudan University Shanghai Cancer Center, Department of Oncology, Shanghai Medical College, Fudan University, Shanghai, China; ^3^ Emergency Department, 305 Hospital of People’s Liberation Army, Beijing, China

**Keywords:** PDAC, tumor microenvironment, cell–cell communication, integrin, prognostic signature

## Abstract

**Background:**

Pancreatic ductal adenocarcinoma (PDAC) is characterized by intensive stromal involvement and heterogeneity. Pancreatic cancer cells interact with the surrounding tumor microenvironment (TME), leading to tumor development, unfavorable prognosis, and therapy resistance. Herein, we aim to clarify a gene network indicative of TME features and find a vulnerability for combating pancreatic cancer.

**Methods:**

Single-cell RNA sequencing data processed by the Seurat package were used to retrieve cell component marker genes (CCMGs). The correlation networks/modules of CCMGs were determined by WGCNA. Neural network and risk score models were constructed for prognosis prediction. Cell–cell communication analysis was achieved by NATMI software. The effect of the ITGA2 inhibitor was evaluated *in vivo* by using a *Kras^G12D^*-driven murine pancreatic cancer model.

**Results:**

WGCNA categorized CCMGs into eight gene coexpression networks. TME genes derived from the significant networks were able to stratify PDAC samples into two main TME subclasses with diverse prognoses. Furthermore, we generated a neural network model and risk score model that robustly predicted the prognosis and therapeutic outcomes. A functional enrichment analysis of hub genes governing gene networks revealed a crucial role of cell junction molecule–mediated intercellular communication in PDAC malignancy. The pharmacological inhibition of ITGA2 counteracts the cancer-promoting microenvironment and ameliorates pancreatic lesions *in vivo*.

**Conclusion:**

By utilizing single-cell data and WGCNA to deconvolute the bulk transcriptome, we exploited novel PDAC prognosis–predicting strategies. Targeting the hub gene ITGA2 attenuated tumor development in a PDAC mouse model. These findings may provide novel insights into PDAC therapy.

## Introduction

Pancreatic ductal adenocarcinoma (PDAC) is one of the most aggressive and fatal diseases and accounts for almost 95% of pancreatic malignant cancers. Over the last decade, intense efforts to improve survival rates have failed. Effective treatment for PDAC patients is largely limited by a low early diagnosis rate, high relapse probability, and the therapy-refractory nature of PDAC; as a result, it has the lowest 5-year survival rate among cancer types (1, 2). PDAC is predicted to become the second leading cause of cancer-related death by 2030 (3). Facilitated by advances in high-throughput technologies, understanding the molecular landscape may be beneficial for solving PDAC.

The molecular classification of PDAC based on genome and transcriptome data is helpful to identify clinically relevant gene signatures, actionable genetic variations, and/or prognostic biomarkers (4–8). However, conventional molecular analyses may be inefficient in fully dissecting microenvironment dynamics. One hallmark of PDAC is extensive inclusion of the stroma. The high heterogeneity of cell components within the PDAC stroma makes it difficult to map distinguishable changes into specific microenvironmental components.

One way to resolve this challenge is to apply deconvolution algorithms, which evaluate the relative abundance of well-known cell types (9, 10). The emergence of single-cell RNA sequencing technology enables the assessment of expression profiles at the single-cell level and the discovery of individual cell type–specific gene profiles. Moreover, according to known ligand–receptor pairs, some bioinformatic tools can be used to estimate a possible crosstalk between different cell populations using scRNA-seq data (11, 12). Thus, this technology informs how different cell types cooperate with each other. Nevertheless, the application of this technology is restricted by its high costs. Moreover, in public PDAC single-cell datasets, limited samples have been included (13, 14).

To take advantage of both single-cell and bulk transcriptome assays in PDAC classification, we utilized scRNA-seq data to deconvolute the bulk transcriptome, which resulted in TME-related gene networks. Based on the coexpressed TME genes, we generated outcome predictors based on neural networks and risk scores. Then, by using a cell–cell communication algorithm, an intercellular connection network was determined in the single-cell transcriptome, which could also be extended to bulk gene expression data for prognostic analyses. Ultimately, the factors involved in the cell–cell communication network were tested for the *in vivo* druggable value, emphasizing a tumor-promoting role of the key integrin in PDAC.

## Materials and Methods

### Data Acquisition and Processing

A single-cell RNA sequencing (scRNA-seq) dataset of pancreatic adenocarcinoma samples was obtained from the Genome Sequence Archive (GSA) database under the accession code CRA001160 (13). The count matrix was directly downloaded from the website. Low-quality cells were removed according to the results of the ‘calculateQCMetrics’ function in the ‘Scater’ package. Three PDAC bulk sequencing transcriptome assays, GSE28735 (15), GSE62452 (16), and GSE71729 (17), were obtained from the Gene Expression Omnibus (GEO) database using the R package ‘GEOquery’. The raw data of the PDAC RNA-seq dataset GSE79668 (18) and two single-cell sequencing repositories GSE155698 (19) and GSE156405 (20) were downloaded from the supplementary file on the GEO website. In The Cancer Genome Atlas (TCGA) database, the transcriptome data, genetic copy number variation data, simple nucleotide variation data, and clinical features of pancreatic adenocarcinoma samples were downloaded and integrated through the ‘TCGAbiolinks’ package (21). From the International Cancer Genome Consortium (ICGC) database (22), we downloaded the RNA-seq data of PACA-AU and PACA-CA and array-based gene expression profiling data (exp_array) together with clinical information. To construct a training set, we integrated three RNA-seq datasets PACA-AU, PACA-CA, and GSE79668 into a combined PDAC dataset using the ‘combat’ R package. The samples lacking prognostic information were excluded from the combined PDAC dataset.

### Re-Analysis of Single-Cell RNA-seq Data

The single-cell RNA-seq data were analyzed by the ‘Seurat’ package (23). Firstly, gene counts were converted to log2(TPM+1) values. Then, the top 2,000 variable features were selected to perform PCA dimension reduction, followed by dimension reduction through Uniform Manifold Approximation and Projection (UMAP). Finally, the Seurat clusters were determined by ‘FindNeighbors’ and ‘FindClusters’ functions in the Seurat package. The cellular identity of each cluster was identified by the expression of cell type–specific genes: epithelial cells [EPCAM (24), KRT19 (25)], pancreatic islet [INS (26)], pancreatic acinar cells [CPA1 (27)], immune cells [PTPRC/CD45 (28)], B cells [MS4A1/CD20 (29), CD79A (30)], T cells [CD3E (29)], myeloid cells [ITGAX/CD11C (31)], endothelial cells [CDH5 (32)], and fibroblasts [COL1A2 (33)].

The highly expressed genes in each cell type were calculated by the ‘FindConservedMarkers’ function. Cell component marker genes (CCMGs) were defined as the genes with a fold change >2 and P-value <0.05, and the genes that were assigned with more than one identity were excluded. The TME marker genes (MEMGs) were defined by the CCMGs representing the B cell, T cell, myeloid cell, endothelial cell, and fibroblast.

### Weighted Correlation Network Analysis

Weighted correlation network analysis (WGCNA) was performed through the ‘WGCNA’ R package. CCMGs were used as input genes for WGCNA. Input genes and samples were filtered by the good genes sample test *via* the ‘goodSamplesGenes’ function. The soft thresholding power β was chosen as the lowest power when the scale-free fit R (2) nears 0.85. In this study, β = 5 was selected to construct the scale-free network, generating eight non-gray gene modules. The eigengene values of the gene modules were calculated by the ‘moduleEigengenes’ function.

The prognostic significance of each module was determined by univariate cox regression analyses and Kaplan–Meier analyses using ‘survival’ and ‘survminer’ packages, respectively. The optimal cutoff values were estimated by the R package ‘maxstat’. The hub genes in each module were determined using both intramodular connectivity (kWithin) and module membership (kME) scores. The functional enrichment of hub genes was performed by the ‘enricher’ function within the ‘ClusterProfiler’ package, using gene sets in the Reactome database (msigdbr, version 7.1.1).

### Unsupervised Transcriptome Clustering

A consensus clustering of PDAC transcriptome data was performed *via* the R package ‘CancerSubtypes’ (34), based on the expression of the MEMGs in blue and green modules, under the parameters clusterAlg=“km”, distance=“euclidean”. The samples in each TME cluster were further filtered by the silhouette score calculated by the ‘silhouette_SimilarityMatrix’ function. Gene expression in TME clusters was visualized by the ‘ComplexHeatmap’ package. In the TCGA-PAAD dataset, genetic variations in TME clusters were summarized and visualized by the ‘oncoplot’ function in the ‘maftools’ package.

### Estimation of Tumor-Microenvironmental-Infiltrating Cells 

To quantify the abundance of immune cells and other TME cells, we used the R package ‘quanTIseq’ to deconvolute the RNA-seq data of PDAC samples (10). The tumor immune dysfunction and exclusion (TIDE) algorithm was used to calculate tumor sample–infiltrating myeloid-derived suppressor cells (MDSCs) and predict immunotherapy responsiveness in PDAC patients (35). The python script tidepy-1.3.7 was used to perform the TIDE program.

### Neural Network Model and Risk Score Construction

To construct a prognosis-predicting model, we employed PyTorch to build a five-layer deep neural network (DNN) model (36) based on the expression of MEMGs. To train the DNN model, a randomly selected 2/3 subset of combined PDAC samples was used as a training set. The batch normalization was conducted in each layer. The Relu function was used as the activation function, and the sigmoid function was applied in the output layer. The trained model was applied in the other 1/3 subset of combined PDAC samples for internal testing and also subjected to external testing in other PDAC datasets. The probability value generated by the DNN program was also used in prognostic analyses. Alternatively, another MEMG-based risk model was defined as weighted average expression of MEMGs. The Cox coefficient was used as the weight for each gene. The risk score was established in a combined PDAC dataset and tested in other datasets. Meta-analysis was performed in R using the ‘metafor’ package (37) with the DL (DerSimonian and Laird) model.

### Cell–Cell Communication Analysis

The intercellular cell–cell communication network in single-cell RNA-seq data was constructed by the Network Analysis Toolkit for the Multicellular Interactions (NATMI) (11). The ligand–receptor pairs were restricted to the cell junction molecules within WGCNA hub genes and extracted from a published ligand–receptor interaction list connectomeDB2020 (38). The cell–cell communication score in bulk RNA-seq data was defined as the geometric mean of (TPM_Ligand_/TPM_Ligand_reference_) and (TPM_Receptor_/TPM_Receptor_reference_). The genes used as a reference for each cell type were as follows: tumor cells (EPCAM), endothelial cells (CDH5), fibroblasts (COL1A2), myeloid cells (ITGAM), pancreatic acinar cells (CPA1), pancreatic islet (NEUROD1), B cells (MS4A1), and cytotoxic cells (CD3E).

### Tissue Samples and Immunohistochemistry

A set of tissue microarrays (TMAs) containing 66 PDAC samples purchased from Shanghai Outdo Biotech Co., Ltd. were used for immunohistochemistry (IHC) staining. Patients and their clinical characteristics are shown in **Supplementary Table 1**. This study has been approved by the Ethics Committee of Renji Hospital, Shanghai Jiao Tong University School of Medicine. For IHC analysis, the slide was rehydrated and then immersed in a 3% hydrogen peroxide solution for 15 min. The slide was pretreated by microwave for 25 min in 0.01 mol/L citrate buffer, pH 6.0, at 95°C; and naturally cooled to room temperature. Between each incubation step, the slide was washed with Phosphate Buffered Saline (PBS), pH 7.4. Then, the tissues were incubated overnight at 4°C with a diluted anti-ITGA2 antibody (Abcam, Ab133557). After washing with PBS, the section was visualized using the VECTASTAIN^®^ Elite ABC-HRP Kit, Peroxidase (Vectorlabs, PK-6104) as per the manufacturer’s instructions.

### Immunofluorescence

Sections were deparaffinized and rehydrated in xylene (2 × 15 min) and gradient alcohol (85% and 70%; 5 min each) and rinsed for 5 min in distilled water. Pretreatment occurred in a sodium citrate antigen retrieval solution (pH 6.0) at a sub-boiling temperature for 10 min, standing for 10 min, and then followed by another sub-boiling temperature for 7 min. After cooling to room temperature, the slides were washed in PBS (pH 7.4) for three times (5 min each). The sections were then immersed in 3% H_2_O_2_ and incubated at room temperature for 15 min in a dark place. After washing three times with PBS in a rocker device, the sections were blocked with 3% BSA for 30 min at room temperature and then incubated at 4°C overnight with primary antibodies against ITGA2 (Abcam, Ab133557, 1:2,500). After being washed with PBS for three times, the tissues were incubated with a secondary antibody (Servicebio, GB23303, 1:500) at room temperature for 50 min in a dark condition. Slides were incubated with a TSA-CY3 solution (appropriately diluted with TBST) for 10 min in a dark condition and then washed three times with TBST. After antigen retrieval again, the slides were treated with an FN1 (Servicebio GB13091, 1:300) and its secondary antibody (Servicebio, GB25303, 1:400). Then, the slides were incubated with a spontaneous fluorescence quenching reagent for 5 min and washed under flowing water for 20 min. Nuclei were stained with 4′, 6-diamidino-2-phenylindole (DAPI) at room temperature for 10 min and treated with an anti-fade mounting medium after washing with PBS.

### Mice and Treatment

The *Pdx1-Cre* mice were crossed with *Kras^(LSL-G12D)^* mice (Shanghai Model Organisms Center, Inc., Shanghai, China) to generate mice with the genotype *Pdx1-Cre^+^, Kras^G12D^* (KC). The 12–16-week-old mice were orally treated with E7820 (100 mg/kg bodyweight) once a day, for 15 consecutive days. The mice were sacrificed after management. The pancreas was fixed and subjected to hematoxylin and eosin (H&E) staining. The tumoral lesions within the pancreas were diagnosed and statistically analyzed. The fixed mice pancreas specimens were also subjected to immunohistochemical staining with primary antibodies, including anti-CK19 (Servicebio, GB12197), anti-Ki67 (Servicebio, GB111141), anti-αSMA (Servicebio, GB13044), anti-CD31 (Servicebio, GB113151), and anti- Gr1 (Servicebio, GB11229). The Alcian blue staining was performed using the Alcian blue staining kit (Servicebio, GP1040). All animal experiments were approved by the Institutional Animal Care and Use Committee at the Renji Hospital, Shanghai Jiao Tong University School of Medicine.

### Cell Culture and Viability Assay

The PDAC cell lines SW1990 and PANC1 were acquired from the American Type Culture Collection [American Type Culture Collection (ATCC), Manassas, VA, United States], and were maintained at 37°C in 5% CO_2_ in Dulbecco’s modified Eagle medium supplemented with 10% fetal bovine serum. Cells were seeded at 1,000 cells in 200 µl of DMEM per well in 96-well plates. At the indicated time points, 20 µl of the Cell Counting Kit-8 reagent (Beyotime, C0039) was added to each well and incubated at 37°C for 3 h. The absorbance was measured by a spectrophotometer at 450 nm with a reference wavelength of 600 nm.

### Western Blot

Cells were lysed by Radioimmunoprecipitation assay (RIPA) buffer (Thermo Fisher Scientific Inc., 89901) with a protease inhibitor cocktail (F. Hoffmann-La Roche Ltd., 05892970001) and phosphatase inhibitor cocktail (F. Hoffmann-La Roche Ltd., 04906845001). The lysates were clarified by centrifugation at 12 000 g for 20 min at 4°C. Protein concentrations were measured by the BCA protein assay kit (Thermo Fisher Scientific Inc., 23225) and the samples were boiled with loading buffer. Protein samples (50–150 μg) were separated through SDS-PAGE and then transferred to a nitrocellulose filter membrane (Pall Corporation) blocked and incubated with the primary antibodies. After washing with TBST three times, the blots were incubated with the IRDye 800CW Secondary Antibody (licor, 926-32211) and visualized by the Odyssey Sa Infrared Imaging System (LI-COR).

### Real-Time PCR

Total RNA from cells were extracted through the RNAiso Plus kit (Takara Bio Inc., Beijing, China). The cDNA preparation was finished through the primeScript RT Master kit (Takara Bio Inc., Beijing, China). Real-time PCR was performed by the SYBR Green quantitative PCR kit (Life Technology) using the 7500 Real-Time PCR System or ViiA7 System (AB Applied Biosystems, Shanghai, China). The primers include human ITGA2-F: GGCTGGCCCAGAGTTTACAT, human ITGA2-R: ATCGCCCCCTCTCCTAACTT, human GAPDH-F: CATGAGAAGTATGACAACAGCCT, and human GAPDH-R: AGTCCTTCCACGATACCAAAGT.

## Results

### Weighted Gene Coexpression Network Analysis Yielded Eight Cell-Marker-Gene Modules

**Figure 1:**
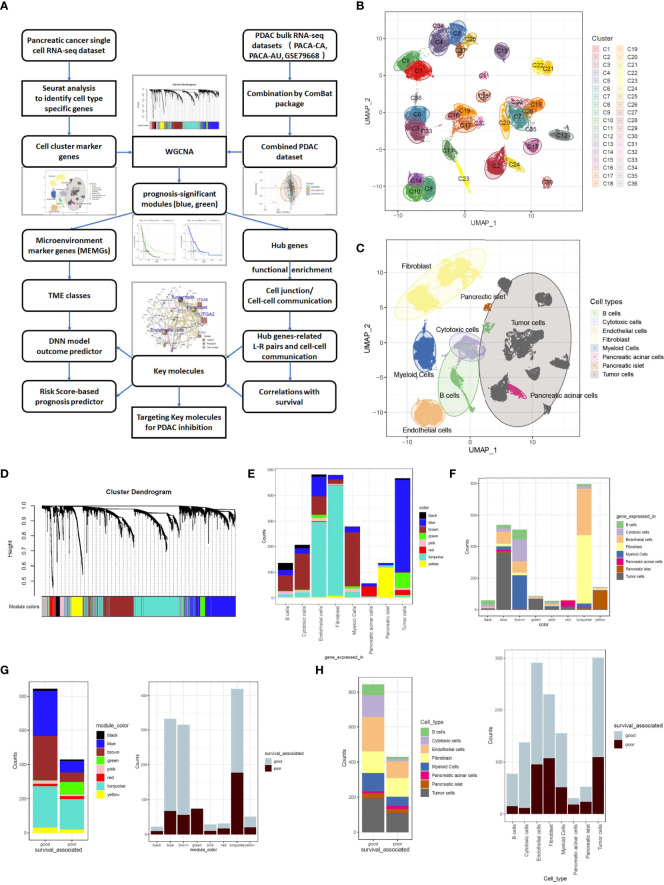
Weighted gene coexpression network analysis (WGCNA) classifies cell component marker genes into eight gene modules in pancreatic cancer. **(A)** The workflow diagram summarizes the study design in this work. **(B, C)** Uniform Manifold Approximation and Projection (UMAP) visualization of pancreatic ductal adenocarcinoma (PDAC) single-cell sequencing data exhibits the cell clusters discovered by Seurat analysis **(B)** and the cellular identity of each cluster **(C)**. The cell type of each cluster was distinguished through the expression of well-documented markers (shown in **Supplementary Figure 1A**) and grouped into eight major components. **(D)** Cell component marker genes found in PDAC single-cell dataset were subjected to WGCNA in a combined PDAC bulk RNA-sequencing dataset. In this process, eight gene-network modules were detected. **(E)** Stacked barplots show the cell origins of the genes constituting each module. **(F)** Distribution of different cell component marker genes in the gene modules. **(G, H)** Barplots show the prognostic significant genes in every gene module **(G)** and each cell type **(H)**. The prognosis significance was determined by Cox regression analysis.

PDAC is dominated by intricate TME cells. To evaluate the prognostic importance of TME variations and key molecular events underlying tumor cell–TME interactions, a set of cell cluster marker genes was defined based on PDAC single-cell RNA-sequencing data. Then, the cell cluster marker genes were used to gauge the TME status in bulk sequencing data to mine prognostic prediction systems in PDAC patients (**Figure 1A**). A single-cell RNA-seq dataset CRA001160 was analyzed by Seurat software, and the dimensions were reduced by UMAP methods to obtain consensus clusters (**Figure 1B**). According to the expression of a handful of well-documented cell type–specific genes (**Supplementary Figure 1A**), we distinguished the cellular identity of each cluster and integrated them into eight major components, including B cells, cytotoxic cells, endothelial cells, fibroblasts, myeloid cells, acinar cells, islet cells, and tumor cells (**Figure 1C**; **Supplementary Figure 1B**). CCMGs were defined as nonoverlapping genes with Fold Change >2 and P-value<0.05 (**Supplementary Table 2**; **Supplementary Figure 1C**). The universality of the defined cell markers was validated by two external scRNA-seq datasets (**Supplementary Figure 2A**). The genes representing B cells, T cells, myeloid cells, endothelial cells, and fibroblasts were then selected and defined as TME marker genes (MEMGs).

To generate a training set for deciphering links between the TME status and prognosis, we merged three cohorts of PDAC bulk RNA-sequencing datasets (PACA-CA, PACA-AU and GSE79668) into one combined PDAC RNA-seq dataset (**Supplementary Table 3**). The ‘Combat’ function was used to eliminate batch effects (**Supplementary Figure 3A**). The CCMGs derived from single-cell data were used as input genes for WGCNA in the combined PDAC dataset under a soft threshold of 0.85, resulting in eight nongrayed gene modules (**Figure 1D**; **Supplementary Figures 3B, C**). When tracing each gene in every module back to its represented cell type, it is clear that all gene modules contained genes from more than one cell type (**Figure 1E**), and the cell type constitution of gene modules was also highly heterogeneous (**Figure 1F**). Tumor cell-derived marker genes were enriched and dominant in the blue and green models, while the turquoise model was mainly composed of endothelial cells and fibroblast-derived genes. CCMGs were tested for their prognostic potential by Cox regression analyses. According to Cox regression results, the CCMGs were classified into good (HR<1, p<0.05) and poor (HR>1, p<0.05) outcome-associated genes (**Supplementary Table 4**). Prognostically significant genes existed in every gene module (**Figure 1G**) and all cell types (**Figure 1H**). The genes indicating good survival were dominant in all modules except the green and red modules.

### MEMGs Classify PDAC Samples Into Prognosis-Related Subtypes

**Figure 2:**
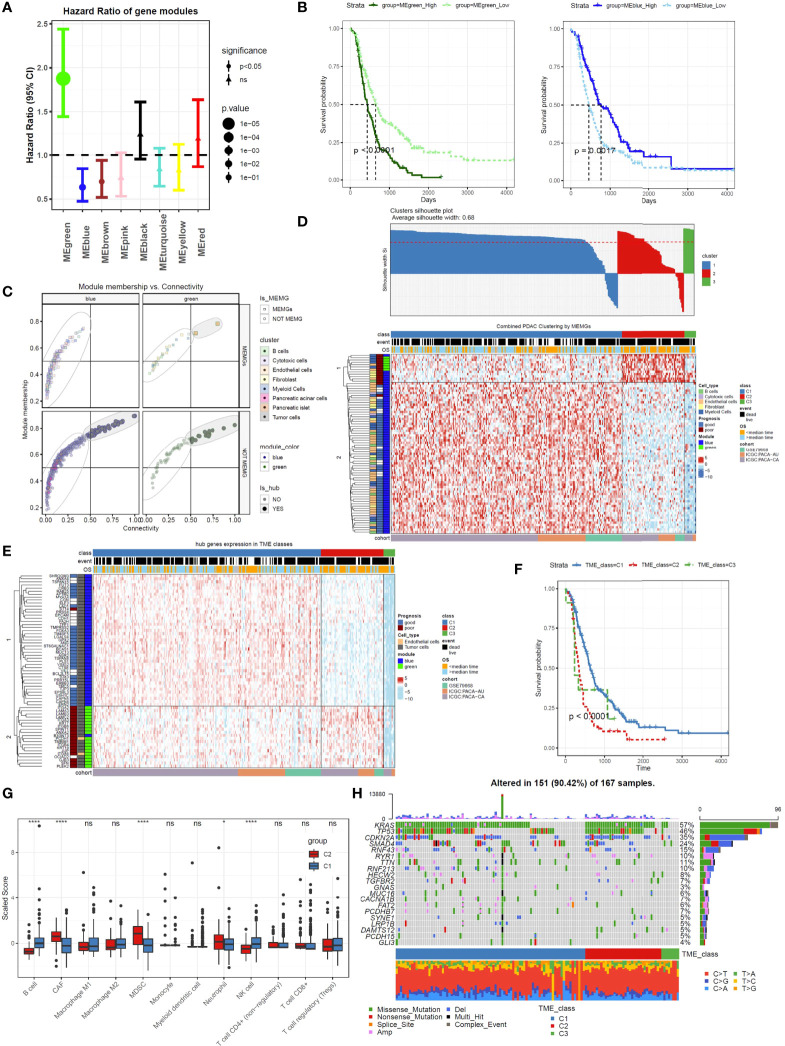
Tumor microenvironment marker genes (MEMGs) stratify PDAC patients into subtypes with distinct outcomes. **(A)** The eigengene (ME) values for the gene modules were calculated in PDAC samples using the transcriptome data; then, the Cox regression analyses were performed to estimate the hazard ratio (HR). Forest plot shows the HR and and 95% confidence interval (95% CI). The optimal cutoff value in each module was estimated by the maxstat function. **(B)** Kaplan–Meier curves indicate that the eigengene values of green and blue modules are correlated with poor and favorite prognosis, respectively, P-values were evaluated by a log-rank tests. **(C)** MEMGs and hub genes in the prognosis-related modules (blue and green modules) were defined by the expression in cell components, module membership, and intramodular connectivity. **(D)** MEMGs stratify PDAC patients into three TME classes by a consensus cluster algorithm. Different expressed MEMGs among subclasses were shown by a heatmap. **(E)** The heatmap shows the expression pattern of hub genes in different TME classes. **(F)** Kaplan–Meier curves show the variations of survival rates among TME classes. Statistical analysis was taken by the log-rank test. **(G)** Relative abundance and differentiations of tumor environmental cells in two major TME classes. The abundance of these cells was estimated by the quanTIseq algorithm or tumor immune dysfunction and exclusion (TIDE) methods. Differences were tested by Student’s t-test, ****P < 0.0001, *P < 0.05, ns: not significant. **(H)** The waterflow plot displays recurrent genetic variations in each TME class.

To understand the prognostic impacts of gene coexpression networks, the module eigengene (ME) values were calculated for Cox regression analysis of each module (**Supplementary Table 5**). Cox regression and Kaplan–Meier analyses under optimal cutoff values revealed that high MEgreen values and low MEblue values inferred unfavorable outcomes (**Figures 2A, B**). In these two modules, the genes with high module membership (MM) values and intramodular connectivity values (MM>0.5 and connectivity>0.5) were defined as hub genes (**Supplementary Table 6**). All 63 hub genes, excluding 2 endothelial genes, in the two modules were derived from tumor cells (**Figure 2C**), which is in agreement with the notion that tumor cells provide the driving force in shaping the TME.

To categorize PDAC patients with respect to TME heterogeneity, we used MEMGs in the green and blue modules to classify the PDAC patients. After filtering samples by silhouette score (silhouette width>0), the remaining samples were assigned to three TME classes (**Figure 2D**). The vast majority of patients fell into the C1 and C2 classes. Most poor-prognostic MEMGs and green module–derived MEMGs were highly expressed in the TME C2 class. For the hub genes, those in the blue module were upregulated in the TME C1 class, while the green genes were highly expressed in the TME C2 class (**Figure 2E**). Importantly, the patients in the C1 and C2 classes were divergent in overall survival rates (**Figure 2F**). To test this finding in the external cohort, the same consensus clustering method was applied in the TCGA-PAAD dataset, resulting in a similar result (**Supplementary Figure 4A**). These results demonstrated that distinct TME statuses were related to patient outcomes. To compare TME components between the main TME classes, we used the ‘quanTIseq’ and ‘TIDE’ deconvolution methods to estimate the abundance of some crucial tumor-infiltrating cells. The results showed that the C1 class was infiltrated by more cytotoxic cells, such as NK cells and CD8^+^ T cells. In contrast, the C2 class was enriched in cancer-associated fibroblasts (CAFs) and immunosuppressive MDSCs (**Figure 2G**). To assess the genetic mutations of the TME classes, the gene variation landscape was mapped. The results showed that the incidence of the recurrent mutations *Kras* and *TP53* was higher in the C2 class (**Figure 2H**).

### Establishment of a Neural Network and Risk Score Model for Predicting Overall Survival and Chemoresponsiveness

**Figure 3:**
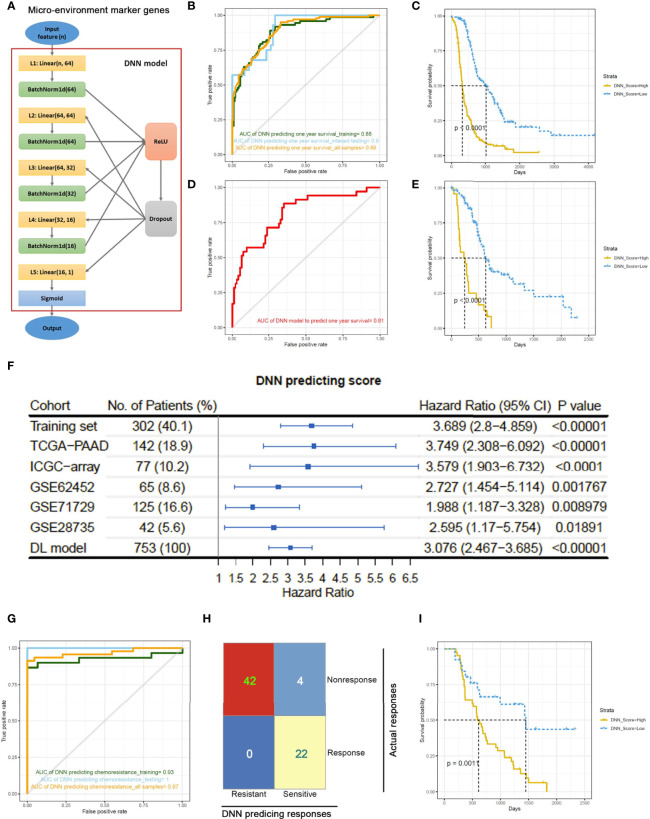
A deep neural network (DNN) model predicts outcomes of PDAC patients based on the expression of MEMGs. **(A)** The framework of the deep neural network (DNN) containing five layers. **(B)** The samples in a combined PDAC dataset were randomly divided into training groups (2/3) and internal testing groups (1/3). The DNN model was trained in training samples by 400 iterations and tested by internal testing samples and all samples. ROC curves show the 1-year survival predicting accuracies in each group. The area under the curve (AUC) values were shown. **(C)** The DNN probability score generated by DNN predictor in the all-sample-testing process was correlated with overall survival of patients. The Kaplan–Meier plot shows the survival rates, and statistical significance was tested by log-rank test. **(D, E)** The prognostic capability of DNN model was examined in the external testing set. The ROC curve **(D)** and Kaplan–Meier plot stratified by DNN scores **(E)** were shown. **(F)** Systematical cox analyses estimate the HR of DNN scores in multiple PDAC datasets from TCGA, ICGC, or GEO databases. The meta-analysis was performed using DL (DerSimonian and Laird) model to estimate the general prognostic effect of DNN score in pancreatic cancer patients. **(G)** The DNN framework was also trained to be a chemotherapy response predictor using a cohort of PDAC patients undergone chemotherapy. ROC curves show the AUC values using a DNN model to predict chemotherapeutic responsiveness in training, testing or all samples. **(H)** A confusion matrix demonstrates the accuracy of DNN model in predicting chemo-response. **(I)** DNN score correlates with patient’s survival after chemotherapy. The survival rates were shown in Kaplan–Meier plot. The differentiation was tested by log-rank test.

To utilize the prognosis-significant gene modules in predicting the overall survival of PDAC patients, a deep neural network (DNN) model was constructed based on the PyTorch platform (36). The DNN model contained five layers and used the blue/green module-derived MEMGs as input (**Figure 3A**). First, the DNN model was trained using 2/3 randomly selected samples in the combined PDAC dataset. Then, the remaining 1/3 of the samples were used as the internal testing set. Since the median survival time is close to 1 year, we applied this model to predict 1-year survival. Consequently, we obtained an AUC=0.88 in the training set and an AUC=0.9 in the testing set (**Figure 3B**). Kaplan–Meier survival analysis of the DNN predictor–derived probability score revealed that the patients with higher scores had shorter survival times (**Figure 3C**). In an external testing set, the TCGA-PAAD dataset, the DNN model was also successful in predicting patient outcomes (AUC=0.81; Kaplan–Meier survival analysis, p<0.0001) (**Figures 3D, E**). Furthermore, meta-analysis was performed to review the prognostic efficiency of the DNN probability score in independent datasets. The results showed that the DNN score was associated with poor prognosis in all tested PDAC datasets. Meta-analysis through the DL model resulted in a positive hazard ratio (HR=3.076, p<0.00001), demonstrating a general prognostic effect of DNN scores (**Figure 3F**). We also utilized the DNN model to predict the chemosensitivity of PDAC patients. In an ICGC subdataset, 68 PDAC patients who underwent chemotherapy were selected for DNN model training, and the trained model accurately predicted the chemoresponsiveness of patients (**Figures 3G, H**; **Supplementary Table 7**). Concurrently, the DNN probability score stratified PDAC patients who received chemotherapy into two groups with distinct survival (**Figure 3I**).

**Figure 4:**
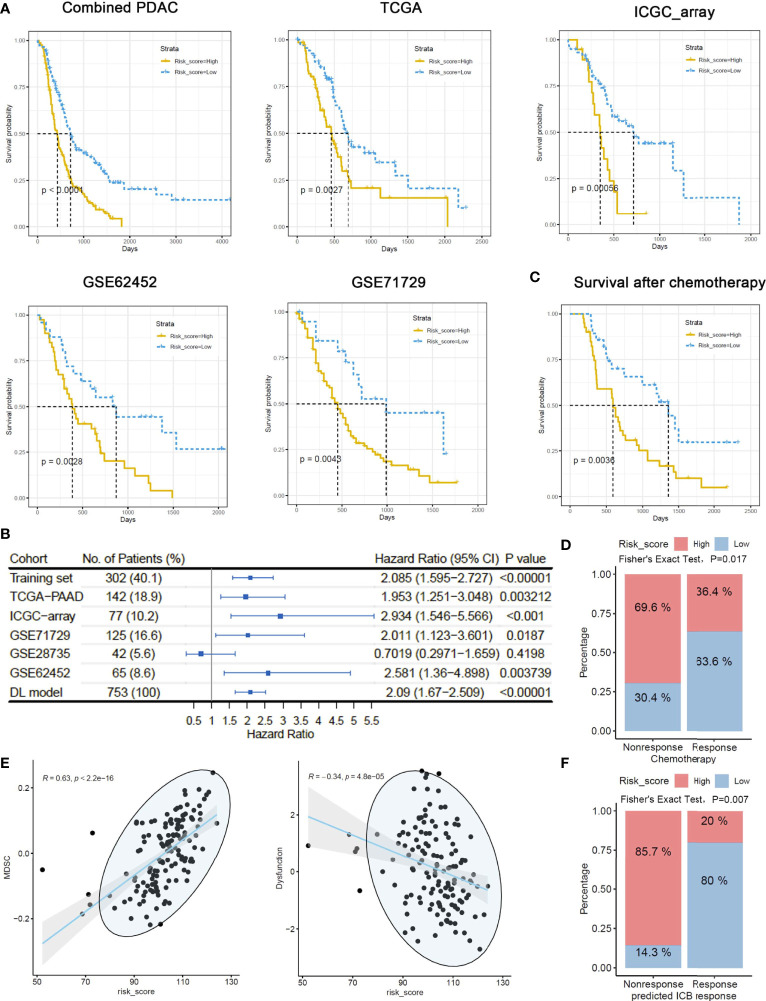
MEMG-based risk score infers therapy efficiencies in pancreatic cancer patients. **(A)** A risk score algorithm based on the expression pattern of MEMGs was established in a combined PDAC (training) dataset and tested for its prognostic relationship in the multiple testing sets. **(B)** The HRs of risk scores in different PDAC datasets were shown, and a meta-analysis with the DL model was performed to estimate the overall effect. **(C)** A Kaplan–Meier plot shows the correlation of the risk score with PDAC patients’ survival after chemotherapy, tested by the log-rank test. **(D)** Correlation between risk score levels and actual responses. The significance was tested by Fisher’s exact test. **(E)** Scatterplots show the correlation of risk score with the TIDE-calculating MDSC abundance and dysfunction level. Correlations were analyzed by the Spearman method. **(F)** Associations of the risk score with TIDE-estimating immune checkpoint blockage therapy effectiveness. The significance was tested by Fisher’s exact test.

In addition to the DNN model, we also adopted a MEMG-based risk score model in prognosis prediction. The risk score was defined as the weighted average expression of MEMGs. The Cox coefficient was used as the weight (**Supplementary Table 8**). In multiple independent PDAC datasets, a high risk score positively correlated with an unfavorable prognosis (**Figure 4A**). Cox regression analyses and meta-analysis showed that higher risk scores inferred poor outcomes in most individual datasets or pooled effects (**Figure 4B**). Again, the risk score was related to outcomes after chemotherapy (**Figure 4C**). Dimidiated by the maxstat-derived cutoff value, the risk score showed a strong correlation with actual chemoresponses (Fisher’s exact test, P=0.017) (**Figure 4D**). We also found that the MEMG-based risk score positively correlated with MDSC infiltration but negatively correlated with the dysfunction score (**Figure 4E**). Importantly, the risk score level was related to the TIDE-estimated immune checkpoint blockage response (Fisher’s exact test, P=0.007) (**Figure 4F**).

### Cell Junction Molecule-Mediated Cell–Cell Communications Govern the Prognosis-Related Gene Network

**Figure 5:**
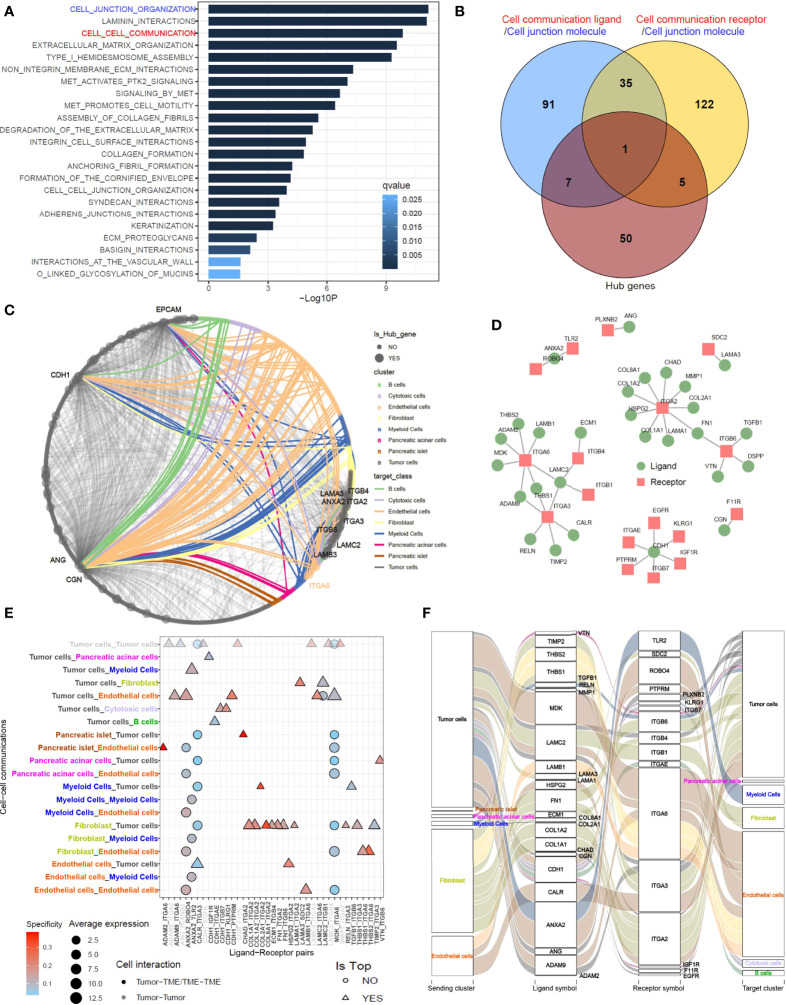
Cell junction molecule–mediating tumor cell–TME communications dominate the prognosis-related gene network. **(A)** Functional enrichment analysis was performed using gene signatures from the Reactome database to unveil the dominant molecular function of hub genes constituting prognosis-related networks. **(B)** Hub genes functioning as cell junction molecules to modulate external cellular communication (ligands/receptors) were selected for the following study. **(C)** A gene correlation network shows the links between the gene pairs initiated from central cell–cell communication mediators (named in the graph) to their closely connected (R > 0.5) genes within modules. Colored edges represent the links involving MEMGs. **(D)** The hub gene–related ligand–receptor pairs were extracted from the connectomeDB2020 database for cell–cell communication prediction in a PDAC single-cell dataset. **(E)** The cell–cell communications mediated by each ligand–receptor pair were determined using Network Analysis Toolkit for the Multicellular Interactions (NATMI) software. The top cell–cell communication pattern bridged by each ligand–receptor pair was noted. **(F)** Alluvial diagram represents the hub gene–associated ligand-receptor pairs engaged in tumor cell–TME communications.

To understand the molecular basis of prognosis-related gene networks, we performed functional enrichment analysis of hub genes using the gene signatures from the REACTOME database. “Cell junction” and “Cell communication” were at the top of the list of enriched molecular function terms (**Figure 5A**; **Supplementary Table 9**). Therefore, the hub genes with cell junction functions that overlapped with either ligands or receptors mediating cellular communication were selected for further analysis (**Figure 5B**). The selected hub genes orchestrated a gene correlation network with convoluted tumor cell–tumor cell or tumor cell–TME connections (**Figure 5C**). The physical ligand–receptor pairs formed by these hub genes were isolated from the connectomeDB2020 database (**Figure 5D**) and subjected to cell–cell communication assays in scRNA-seq datasets. The preliminary results showed that one ligand–receptor pair may modulate more than one cell pair (**Figure 5E**; **Supplementary Figure 5A**). The cell pair with the largest average expression value was considered the top cell–cell connection for each ligand–receptor pair (**Supplementary Table 10**). By matching the ligands/receptors to sending cell types and target cell types according to the top connections, we obtained cell-ligand–receptor–target alluvial plots in which molecules were weighted by average expression and cell types were weighted by summing the average expression of contributing molecules. The networks showed that tumor cells, fibroblasts, and endothelial cells were the most vital cell types engaged in cell–cell communication in pancreatic cancers **(** **Figure 5F**, **Supplementary Figure 5B**).

### Integrins are Key Mediators Critical for Tumor Cell–TME Communications

**Figure 6:**
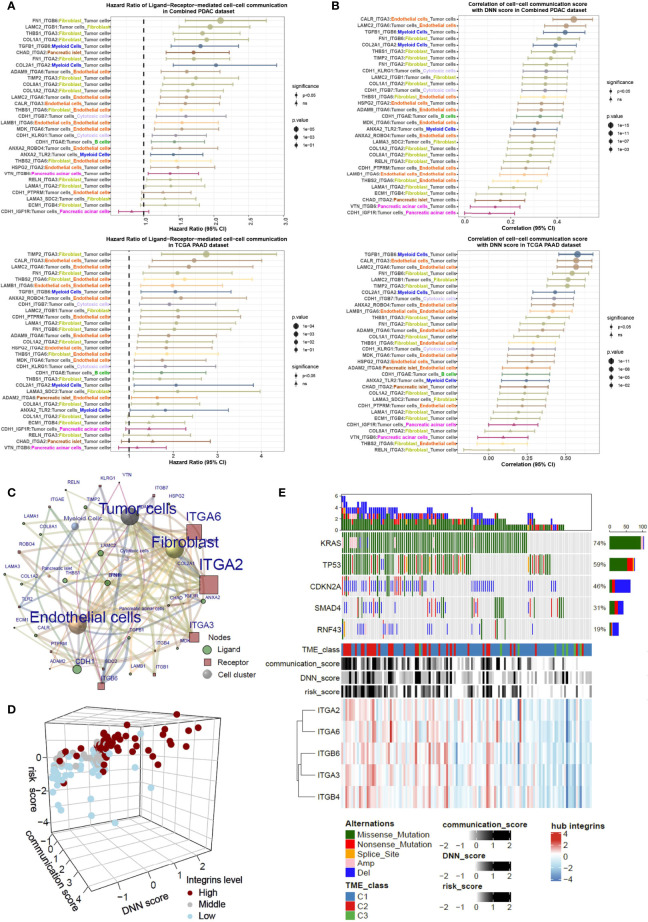
Integrins are key mediators critical for tumor cell-fibroblast and tumor cell-endothelial cell communications. **(A)** The HRs of cell–cell communication scores of PDAC patients in both the combined PDAC dataset and TCGA dataset, analyzed by the Cox regression model. **(B)** Correlation of the cell–cell communication score with DNN scores in both combined PDAC and TCGA datasets. Forest plots show the correlation coefficients together with the 95% confidence intervals. **(C)** A sending cell–ligand–receptor–target cell network shows the key contributors within it. The notes and edges are weighted by the integrated HR in combined PDAC and TCGA datasets. The result indicates that integrin-mediated tumor cell–fibroblast and tumor cell–endothelial cell communications are dominant in the network. **(D)** Three-dimensional plot shows the relationships with the cell–cell communication score, DNN score, risk score, and expression levels of integrins. **(E)** Waterflow plot indicates the enrichment of genetic variations in pancreatic cancers with high levels of cell–cell communications.

To interrogate the prognostic effect of cell–cell communication linked by a particular ligand–receptor pair, we calculated the cell–cell communication score in bulk RNA-seq data for Cox regression analyses in the combined PDAC and TCGA-PAAD cohorts (**Figure 6A**). Most ligand–receptor-induced cell–cell connections were markedly correlated with poor prognosis (HR>1, P<0.05). Notably, integrin-mediated tumor cell-fibroblast communication and tumor cell-endothelial cell communication were prioritized in the HR rank. In addition, the majority of cell–cell communication scores were correlated with the DNN model-generated probability score (correlation coefficient > 0, P<0.05) (**Figure 6B**). By integrating the cell–cell communication networks in both the combined PDAC dataset and the TCGA-PAAD dataset, we found that tumor cells, fibroblasts, and endothelial cells were the most significant cellular components. Meanwhile, integrins such as ITGA2 and ITGA6 were the main contributing molecules (**Figure 6C**; **Supplementary Figure 6A**). The scRNA-seq data showed that ITGA2 was mainly expressed in tumor cells, while its ligands COL1A1, COL1A2, COL8A1, FN1, and HSPG2 were expressed in fibroblasts and endothelial cells (**Supplementary Figure 6B**). ITGA2 interacted with COL1A1, COL1A2, COL8A1, FN1, and HSPG2, mediating the tumor cell–fibroblast and tumor cell–endothelial cell connection. In contrast, another hub, ITGA6, was predominantly expressed in endothelial cells (**Supplementary Figure 6C**). ITGA6 and its ligands mediate the interactions between endothelial cells and tumor cells and other environmental cells. Correlation analyses showed that the mean expression levels of integrins in hub genes were largely in parallel with the cell–cell communication score, DNN probability score, and risk score (**Figure 6D**; **Supplementary Figures 7A, B**). Additionally, PDAC samples highly expressing hub integrins accumulated in the TME C1 class and harbored more driver genetic variations (**Figure 6E**). These results indicated that integrins may be the key mediators in the tumor cell–TME interactions.

### Pharmacological Blockade of ITGA2 Orchestrates Microenvironmental Changes and Limits PDAC Initiation

**Figure 7:**
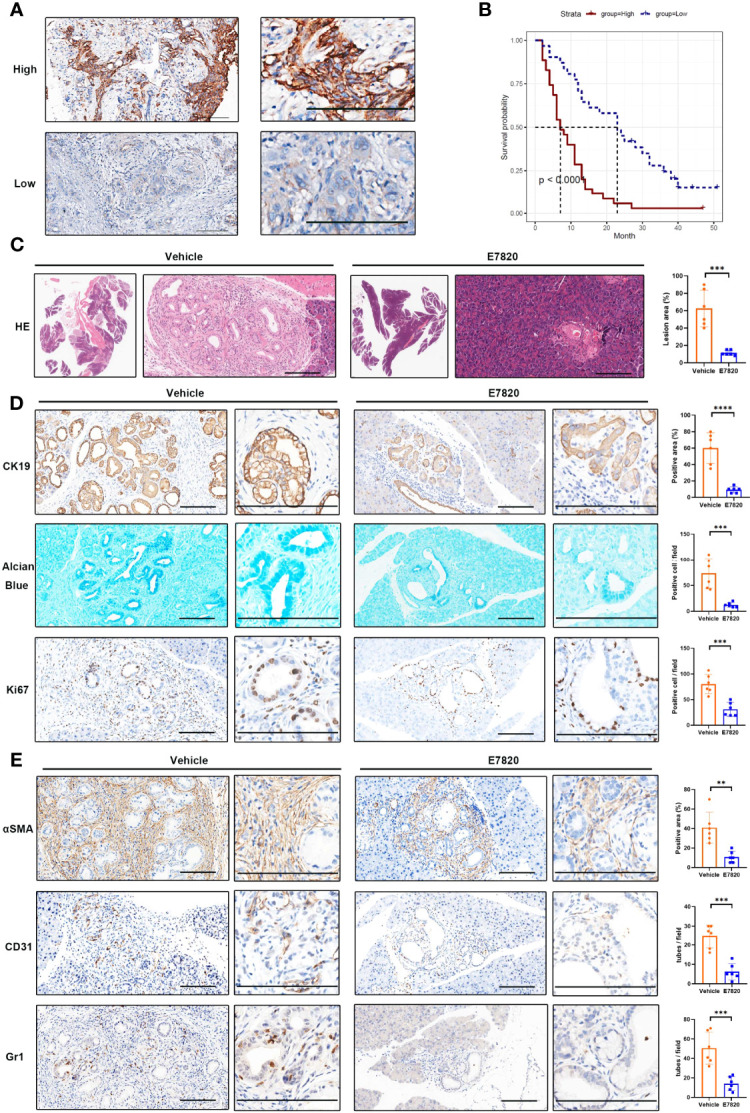
Inhibition of ITGA2 prevents PDAC growth. **(A)** Immunohistochemical analyses of ITGA2 expression in PDAC samples. The representative images of high and low ITGA2 staining in PDAC specimens were shown. **(B)** Kaplan–Meier plots show the overall survival of PDAC patients expressing high and low levels of ITGA2 protein. **(C–E)** *Pdx1-Cre^+^, Kras^G12D^(*KC*)* mice were orally treated with E7820 (100 mg/kg bodyweight) once a day, for 14 consecutive days. **(C)** The H&E staining of mice pancreatic lesions. The percentage of the lesion area was statistically compared between vehicle and E7820 treatment groups, t test, ***P < 0.001, n=6 fields. **(D)** Alcian blue staining of pancreas tissues. The representative images and statistically analysis results of CK19 and Ki67 immunohistochemical staining slides, t test, ***P < 0.001, ****P < 0.0001, n=6 fields. **(E)** Immunohistochemistry analyses of pancreas tissues from vehicle and E7820-treated KC mice by anti-αSMA, anti-CD31, and anti-Gr1 antibodies, t test, **P < 0.01, ***P < 0.001, n=6 fields. Bar=200 μm.

Intrigued by the pivotal roles of integrins in the intercellular network, we sought to choose the most significant tumor cell–expressed integrin, ITGA2, for protein expression and druggable potential testing. In a tissue microarray containing 66 PDAC samples, we performed a immunohistochemical analysis of ITGA2. ITGA2 was shown to be clearly expressed on the membranes of tumor cells (**Figure 7A**). In accordance with the bioinformatics analyses, PDAC patients highly expressing ITGA2 had inferior outcomes (**Figure 7B**). By using an immunofluorescence assay, we detected the colocalization of tumor cell–expressed ITGA2 and fibroblast-expressed FN1, indicating a role of ITGA2 and its ligand in regulating tumor cell–fibroblast interactions (**Supplementary Figure 8A**). To explore whether and to what extent targeting ITGA2 influences PDAC development, we employed *Pdx1-Cre^+^, Kras^G12D^* (KC) mice for *in vivo* inhibitor management. The mice were orally treated with the ITGA2 inhibitor E7820 (39), and the affected pancreas area was quantified. It appeared that E7820 essentially diminished pancreatic lesions without profoundly influencing body weights (**Figure 7C**; **Supplementary Figure 9A**). The efficiency of E7820 treatment was also revealed by the detection of the ductal biomarker CK19, mucin content (Alcian blue staining), and proliferation marker Ki67 (**Figure 7D**). In cultured PDAC cells, E7820 suppressed the mRNA and protein expression of ITGA2 (**Supplementary Figures 9B, C**), in accordance with the molecular mechanism of this inhibitor (40). Importantly, E7820 did not significantly alter the proliferation rate of PDAC cells *in vitro* (**Supplementary Figure 9D**), suggesting that the effects of this compound may depend on the microenvironment. Furthermore, in the mouse model, the pancreatic lesion area of treated mice contained fewer αSMA-positive fibroblasts, CD31-positive microvessels, and Gr1-positive MDSCs (**Figure 7E**). Collectively, our *in vivo* pharmacology experiment revealed that targeting the key molecule in the cell communication network reshaped the tumor-promoting microenvironment and led to growth defects in PDAC.

## Discussion

In high-throughput transcriptome assays, separating gene signatures by different cellular compartments is always an important issue (41). It becomes more challenging in the bulk expression assays of PDAC tissues since pancreatic cancer includes extremely high levels of noncancerous cells. To address this issue, previous studies have developed bioinformatics approaches for the deconvolution of bulk PDAC samples. For example, Moffitt et al. used a method called virtual microdissection to digitally separate PDAC tissue signatures into tumor cell and stromal compartments and stratified samples into tumor cell–based or stroma-based clusters (17). In recent years, the emergence of single-cell RNA sequencing technology may solve the above concern. However, the wide application of this method is restricted by costs. Public access to scRNA-seq data is also limited. Therefore, it is difficult to perform large-scale data mining through scRNA-seq data. To extend the usage of single-cell transcriptome data, in this work, we first extracted cell type-specific information by generating gene sets representing each cellular component. In the second step, the cell-type marker genes were grouped by the WGCNA algorithm for constructing gene coexpression networks. Unlike previous methodologies, our pipeline used WGCNA-derived networks for further classification and prognostic model construction.

After the WGCNA of the CCMGs, we obtained eight nongray gene modules, representing eight gene coexpression networks. Every single module/network was constituted by the genes derived from several cell types, indicating that coexpression could occur between genes expressed in separated cellular components. Therefore, a gene coexpression network may contain information regarding regulatory events across cell types. Here, we mainly focused on the two prognosis-related gene networks, green and blue (**Figures 2A, B**). According to the concepts of WGCNA, the hub genes within the coexpression network may play central roles in the functional group (15). Therefore, it is reasonable for us to assume that the hub genes of these two networks are responsible for the clinical behaviors of PDAC. Because the majority of hub genes could be traced back to tumor cells (**Figure 2C**), our findings reinforce the notion that intrinsic tumor genes shape the microenvironment and regulate tumor development and therapeutic responses (42–44).

In the context of pancreatic cancer, increasing evidence suggests that intrinsic tumor factors may contribute to stromal remodeling (45, 46). One excellent work demonstrated that distinct clones of cancer cells give rise to heterogenous TMEs (47). To screen the key cancer cell–intrinsic molecules, we performed functional enrichment analysis in the hub genes, confirming a strong accumulation of cellular communication and/or cell junction molecules (**Figures 5A, B**). These molecules form a ligand–receptor web, guiding the crosstalk between cancer cells and nontumoral cells (**Figures 5D, E**). Unlike other single-cell studies in pancreatic cancer that analyze general ligand–receptor–directed cell interactions (19, 48, 49), we focused on intercellular crosstalk directed by the hub genes within coexpression networks. Under this prerequisite, the main participators in the network were fibroblasts and endothelial cells, which are frequently connected to tumor cells. The other feature was that we utilized the communication score to analyze the bulk transcriptome and weighted each communication event by the HR. The results highlighted a group of integrins governing the fibroblast–tumor cell or endothelial cell–tumor cell communications that were prioritized in the significant risk factors (**Figure 6A**).

Integrins belong to a family of transmembrane receptors that mediate cell–cell adhesion and cell-to-extracellular matrix (ECM) interactions. On the cell surface, integrins, composed of one α subunit and one β subunit, recognize ECM on one side while linking the cell skeleton and/or intercellular signaling pathways on the other side (50, 51). By attaching to the ECM, integrins respond to these microenvironmental components, transmitting outer signals to the inner compartment. Certain integrins can bind to distinct ECM molecules and vice versa. Therefore, the integrin–ECM interactome is capable of mediating cell–cell communications through either the “one-to-many” or “many-to-one” model (**Figure 5D**; **Supplementary Figure 6A**). For instance, ITGA2 interacts with multiple fibroblast-produced ECM molecules, such as collagens, fibronectins, or laminins (COL1A2, COL1A1, COL8A1, FN1, and LAMA1) (52). These ligand–receptor pairs may be functionally redundant to direct fibroblast–tumor cell interactions. Intriguingly, our finding is consistent with a previous report that also emphasized ECM–integrin-mediated fibroblast–epithelial cell interactions (20).

In this work, the data mining of PDAC scRNA-seq data revealed that ITGA2, ITGA3, ITGB4, and ITGB6 were dominantly expressed in tumor cells. ITGA6, one of the two nontumoral cell–derived hub genes, was mainly expressed in endothelial cells (**Figure 5C**). ITGB1 was overwhelmingly localized in fibroblasts. Some of these integrins have been implicated in the tumorigenesis of PDAC (53–55). The main contributing cell types of some integrins have also been approved by previous work (56, 57). In the hub gene–associated ligand–receptor pairs, ITGA2 was partnered with the greatest number of ligands and was weighted by the highest HR value. Therefore, it is much more likely that ITGA2 is an Achilles’ heel in the cell–cell communication network. Some comprehensive works have shown that cell–cell communication is a potential therapeutic target in pancreatic cancer (56, 58). Targeting ITGA2 may also be an attractive way to inhibit PDAC.

To determine a clinically feasible way to target ITGA2, the ITGA2 inhibitor E7820, which has been used in clinical trials (39, 59–61), was chosen for testing. In PDAC, the effect of E7820 is unclear. To preclinically mimic the *in vivo* performance of this reagent, we used an oncogene-driven spontaneous pancreatic tumor model, in which the oral delivery of E7820 substantially alleviates pancreatic lesions. This *in vivo* pharmacological experiment highlighted the efficiency of the ITGA2 inhibitor. The ITGA2 inhibitor E7820 was initially used as an angiogenesis antagonist that attenuates the tube formation ability of endothelial cells (40). Here, we clearly show that ITGA2 is basically expressed in tumor cells, which was demonstrated by both single-cell transcriptome data and immunohistochemistry detection. Hence, the primary target of E7820 in PDAC was tumor cells rather than endothelial cells. *In vitro* assays showed that similar to cultured endothelial cells, E7820 also reduced the mRNA expression of ITGA2 in PDAC cells, whereas the suppressive effects on cell growth were slight, disproportionate with the inhibitory effect on the tumors *in situ*. Moreover, E7820 considerably reduced the fibroblasts and microvessels around tumor foci. These observations suggested that E7820 shrinks pancreatic tumors in a microenvironment-dependent manner. Targeting ITGA2 may be a prospective strategy to cure PDAC.

## Data Availability Statement

The datasets presented in this study can be found in online repositories. The names of the repository/repositories and accession number(s) can be found in the article/**Supplementary Material**.

## Ethics Statement

The animal study was reviewed and approved by Ethics Committee of Renji Hospital, Shanghai Jiao Tong University School of Medicine.

## Author Contributions

BW, CW, TJ, and YL designed the research, analyzed data, performed experiments, and wrote the manuscript. DW, LT, and LY performed some experiments and provided intellectual inputs. All authors contributed to the article and approved the submitted version.

## Funding

This work was supported by the National Natural Science Foundation of China (82172905, 81972209, 82060041) and Shanghai Natural Science Fund (21ZR1461500).

## Conflict of Interest

The authors declare that the research was conducted in the absence of any commercial or financial relationships that could be construed as a potential conflict of interest.

## Publisher’s Note

All claims expressed in this article are solely those of the authors and do not necessarily represent those of their affiliated organizations, or those of the publisher, the editors and the reviewers. Any product that may be evaluated in this article, or claim that may be made by its manufacturer, is not guaranteed or endorsed by the publisher.

## Abbreviations

CAF, cancer-associated fibroblasts; CCMG, cell component marker genes; DNN, deep neural network; kME, module membership; kWithin, intramodular connectivity; MDSC, myeloid-derived suppressor cells; ME, module eigengene; MEMGs, tumor microenvironment marker genes; MM, module membership; NATMI, Network Analysis Toolkit for the Multicellular Interactions; PDAC, pancreatic ductal adenocarcinoma; TIDE, tumor immune dysfunction and exclusion; TME, tumor microenvironment; TPMs, transcripts per kilobase million; UMAP, Uniform Manifold Approximation and Projection; WGCNA, weighted correlation network analysis.
