# Supplementary material for: Gene Coexpression Network Characterizing Microenvironmental Heterogeneity and Intercellular Communication in Pancreatic Ductal Adenocarcinoma: Implications of Prognostic Significance and Therapeutic Target
Source: Front Oncol. 2022 Jun 1;12:840474. doi: 10.3389/fonc.2022.840474 (PMC9198606; doi:10.3389/fonc.2022.840474)
Supplement: Supplementary file 1 [file DataSheet_1.pdf]

Supplementary Figures

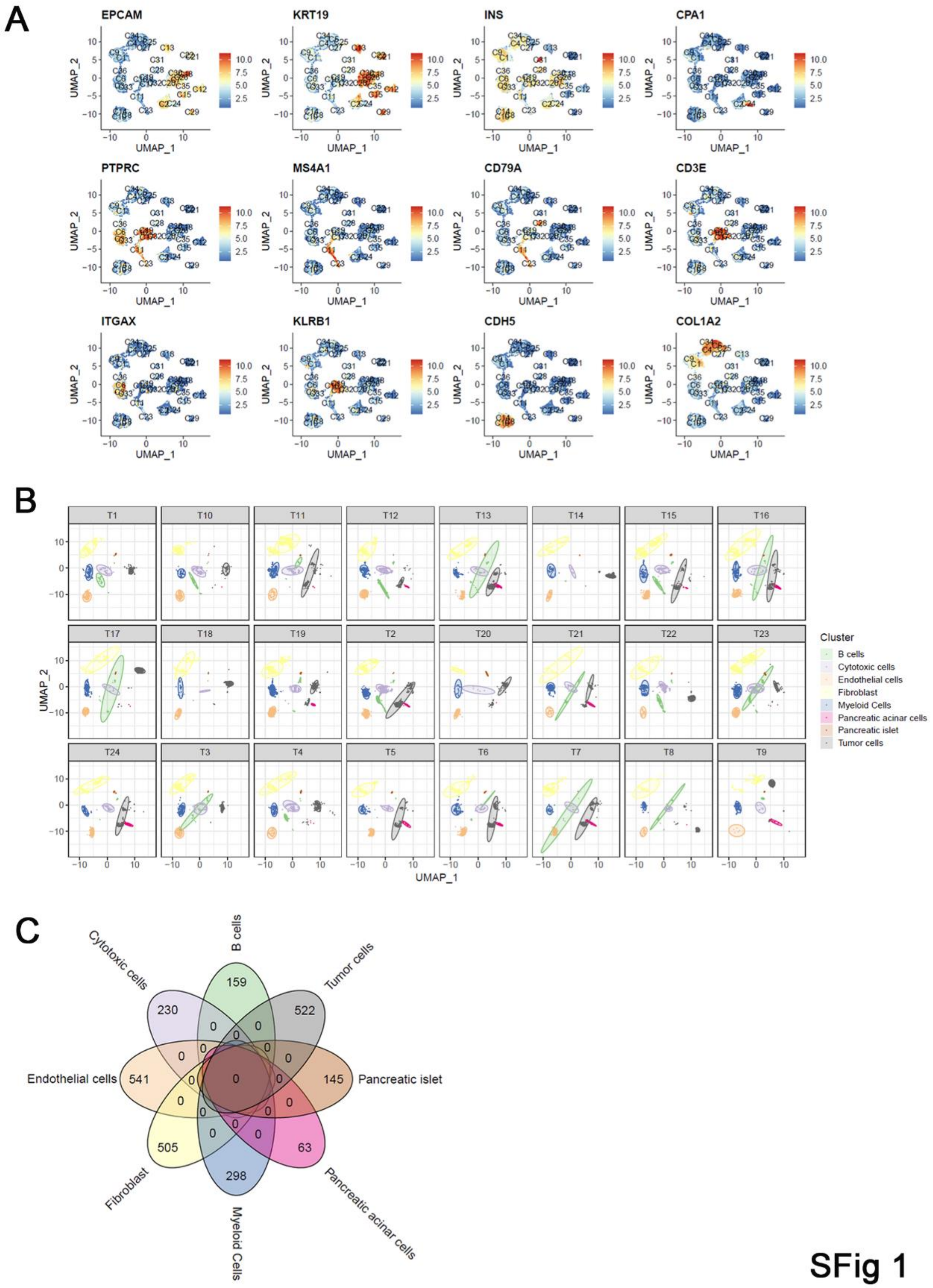

SFig 1

Supplementary Figure 1. Define Cell Component Marker Genes using PDAC single cell RNA-sequencing data

(A) The expression of cell markers in clusters identified by Seurat analysis. Tumor cells: EPCAM, KRT19, pancreatic islet: INS, pancreatic acinar cell: CPA1, immune cells: PTPRC (CD45), B cells: MS4A1(CD20), CD79A, T cells: CD3E, endothelial cells: CDH5, fibroblast: COL1A2. (B) Cell components in individual patients. (C) Non-overlapped Cell Component Marker Genes in eight dominant cell types.

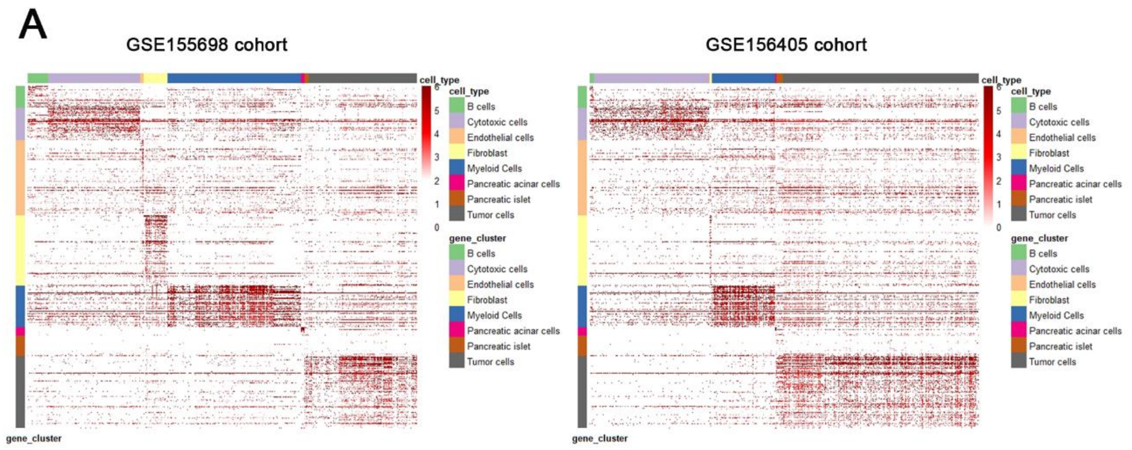

SFig 2

Supplementary Figure 2. External validation of Cell Component Marker Genes

(A) In scRNA-seq datasets GSE155698 and GSE156405, cell types were clustered and identified. The cell type-specific genes in GSE155698 and GSE156405 were compared to CRA001160-derived Cell Component Marker Genes for validation.

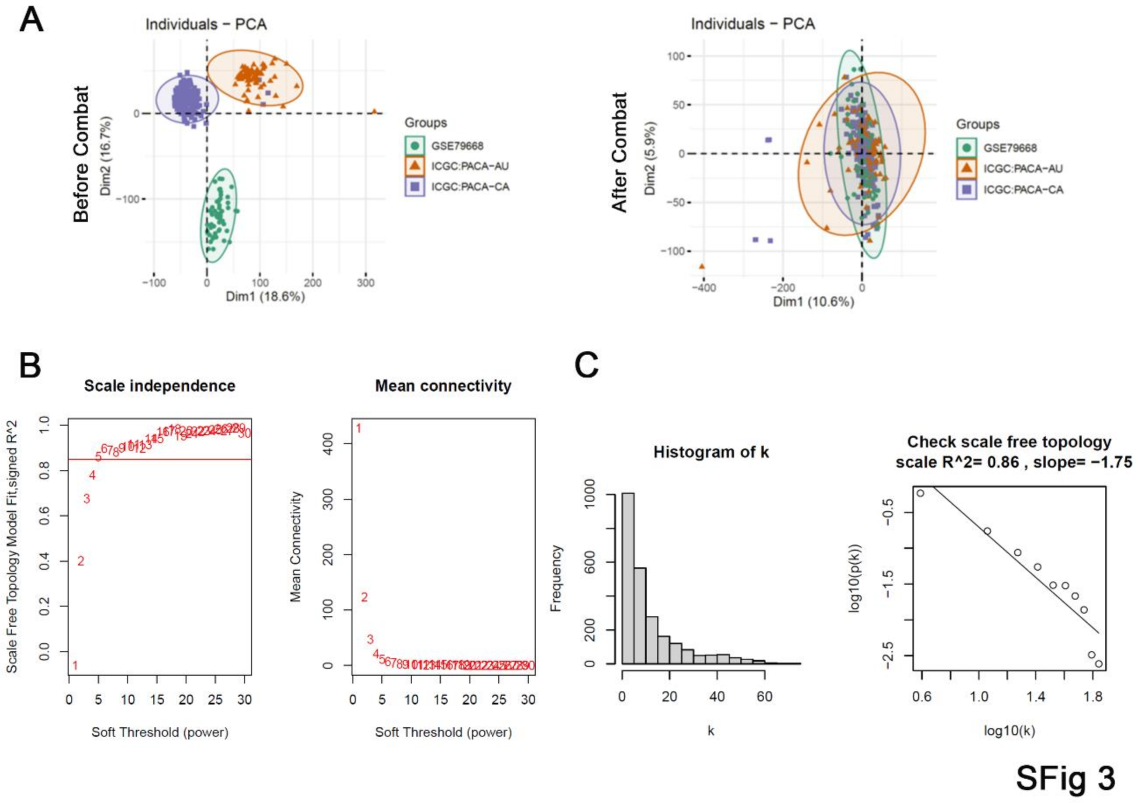

Supplementary Figure 3. WGCNA analysis in a combined PDAC transcriptome dataset

(A) Principal component analysis (PCA) analyses of combined PDAC dataset before and after data integration using combat software. (B) Soft threshold selection based on network topologies and mean connectivity (R-squared cutoff is set to 0.85, β=5). (C) Tests of scale free topology under the selected beta value.

A

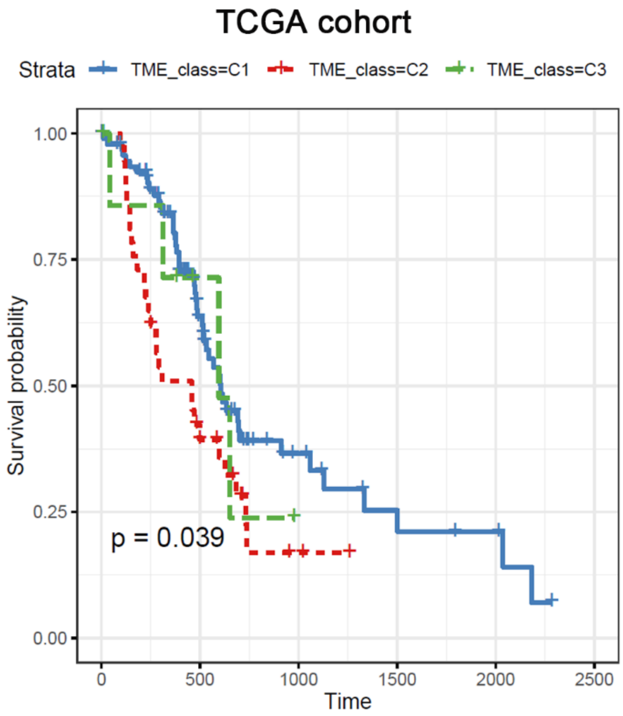

SFig 4

Supplementary Figure 4. The TME classes in TCGA-PAAD dataset are different in survival rates

(A) The patients in TCGA-PAAD cohort were stratified by MEMGs through consensus clustering method and viewed in Kaplan-Meier plot, tested by log-rank test.

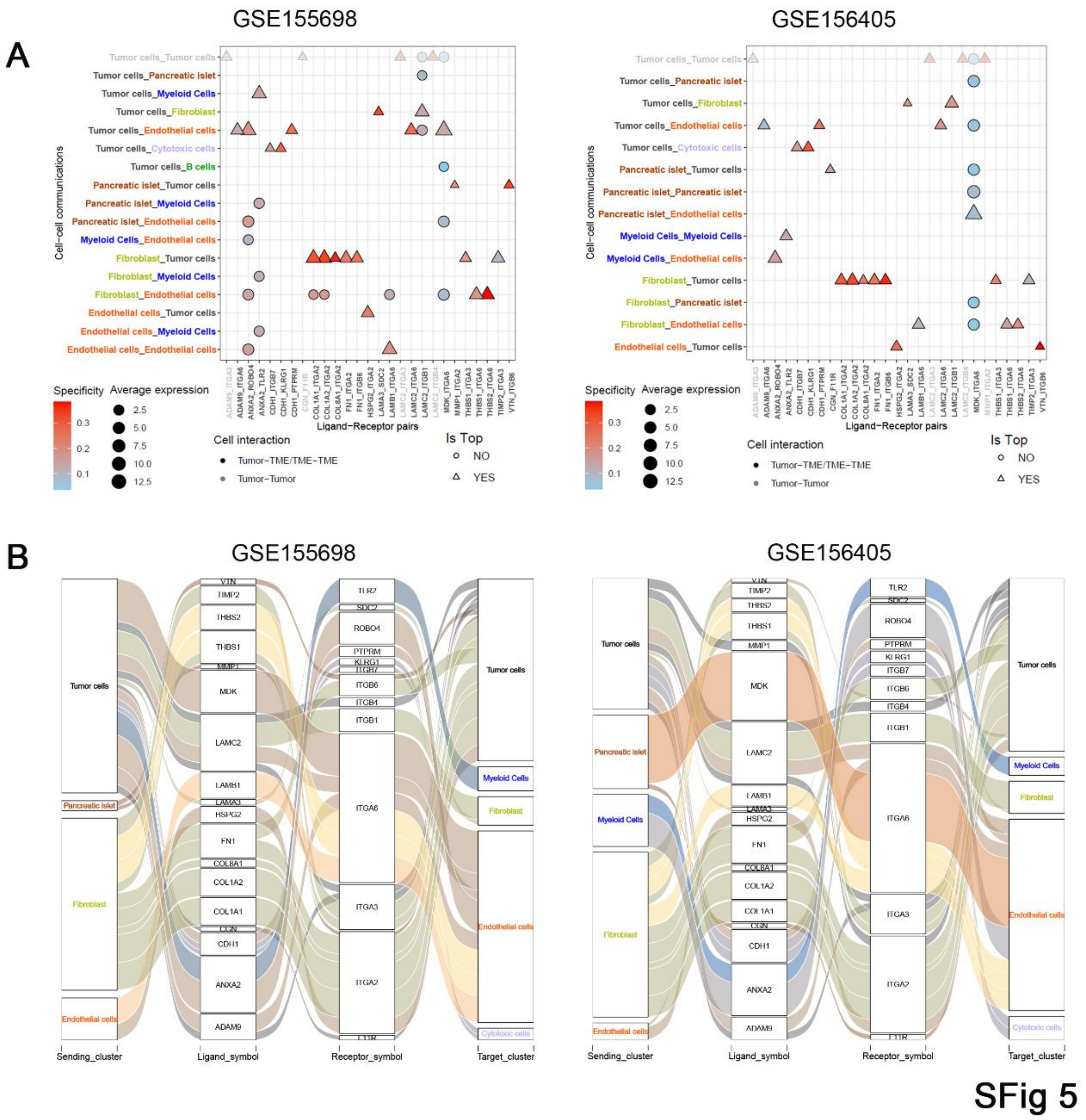

Supplementary Figure 5. Cell junction molecule-mediating cell-cell communications in scRNA-seq datasets

(A-B) In scRNA-seq datasets GSE155698 and GSE156405, cell-cell communications mediated by ligand-receptor pairs were determined using Network Analysis Toolkit for the Multicellular Interactions (NATMI) software. The top cell-cell communication pattern linked by each ligand-receptor pair was noted (A). Alluvial diagram represents the ligand-receptor pairs engaged in tumor cell-TME communications (B).

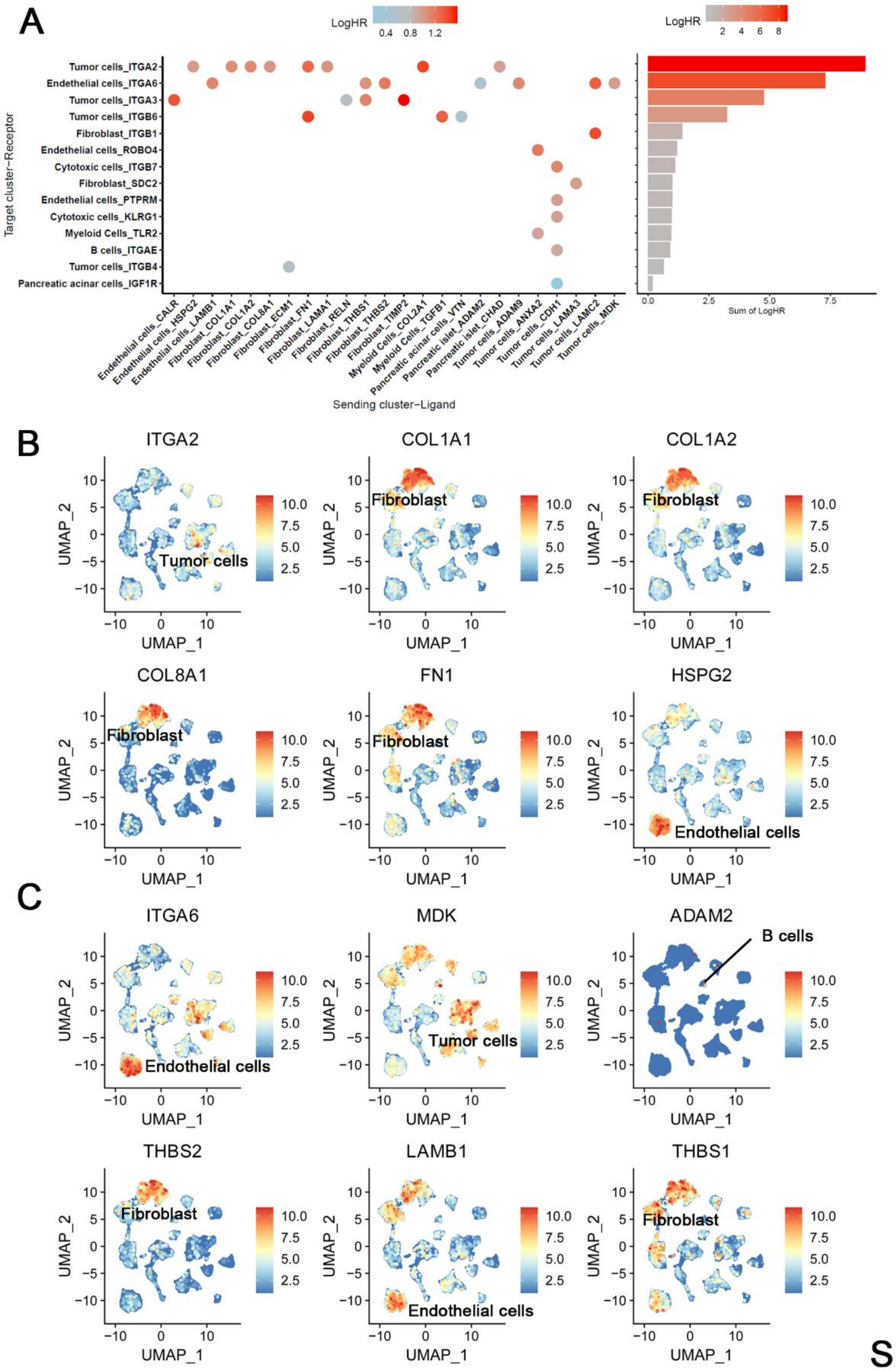

SFig 6

Supplementary Figure 6. The ligand-receptor pairs regulating cell-cell communications

(A) Dotplot shows the integrated hazard ratio of ligand-receptor pairs. (B-C) Featureplots exhibit expression patterns of ITGA2(B), ITGA6(C) and their major ligands.

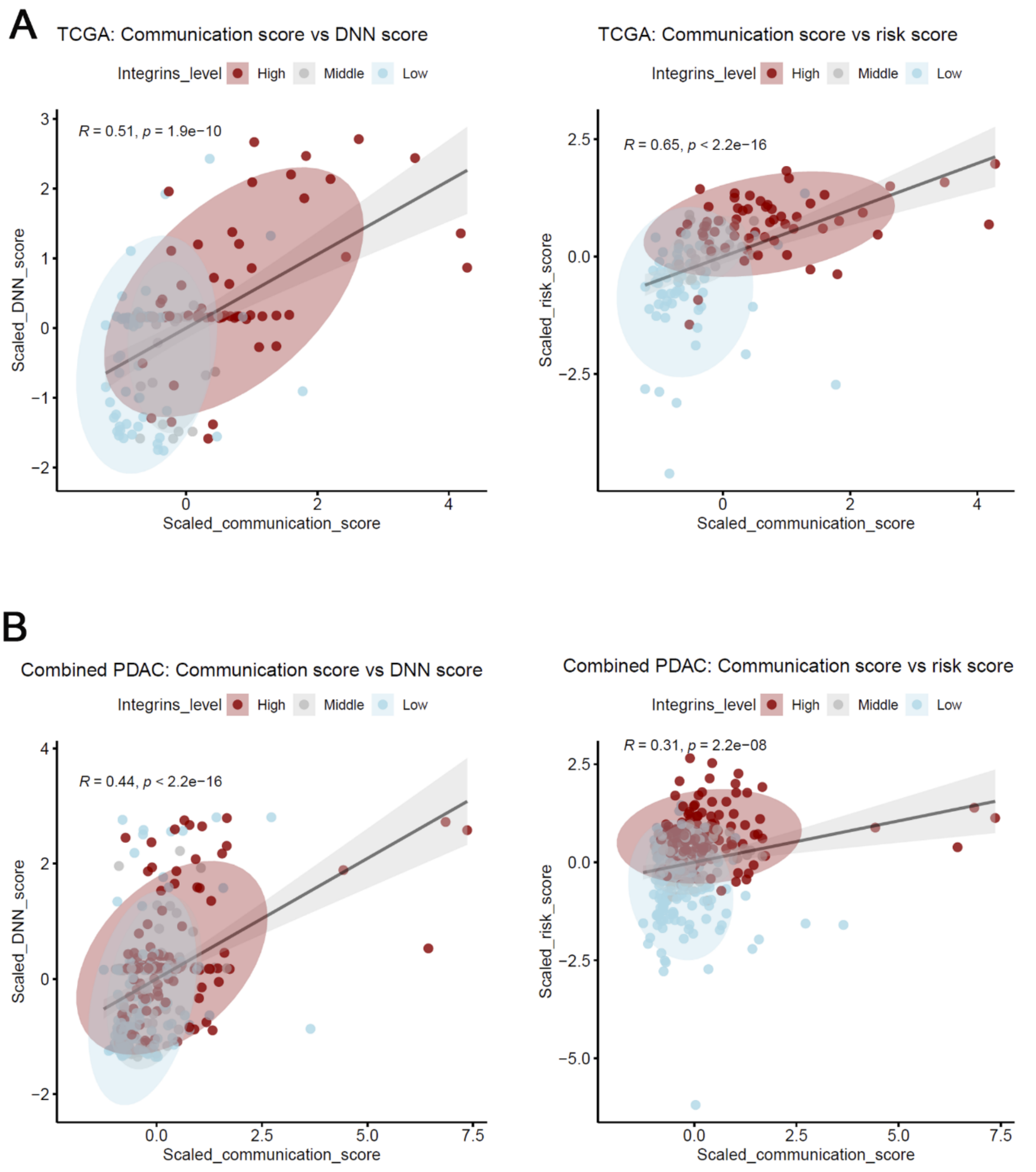

SFig 7

Supplementary Figure 7. Cell-cell communication score in parallel with integrin expression level correlates with DNN score and risk score

(A-B) Scatterplots show the correlation of cell-cell communication score with DNN score and risk score in TCGA-PAAD (A) and combined PDAC (B) datasets.

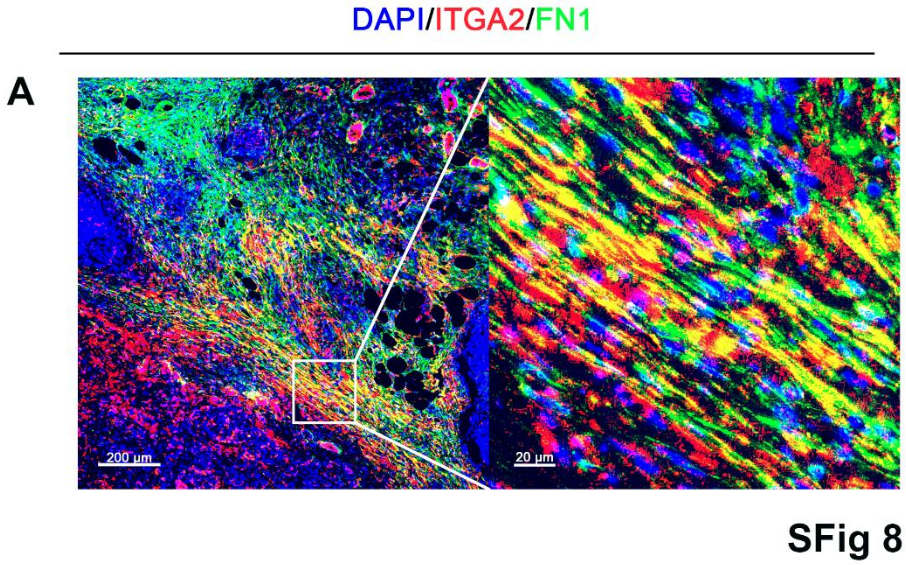

Supplementary Figure 8. Colocalization of ITGA2 and FN1 in PDAC tissue

(A) Immunofluorescence staining of ITGA2 (red) and FN1 (green) in PDAC tissue samples. The yellow color indicates colocalization. Nuclei were visualized with DAPI (blue).

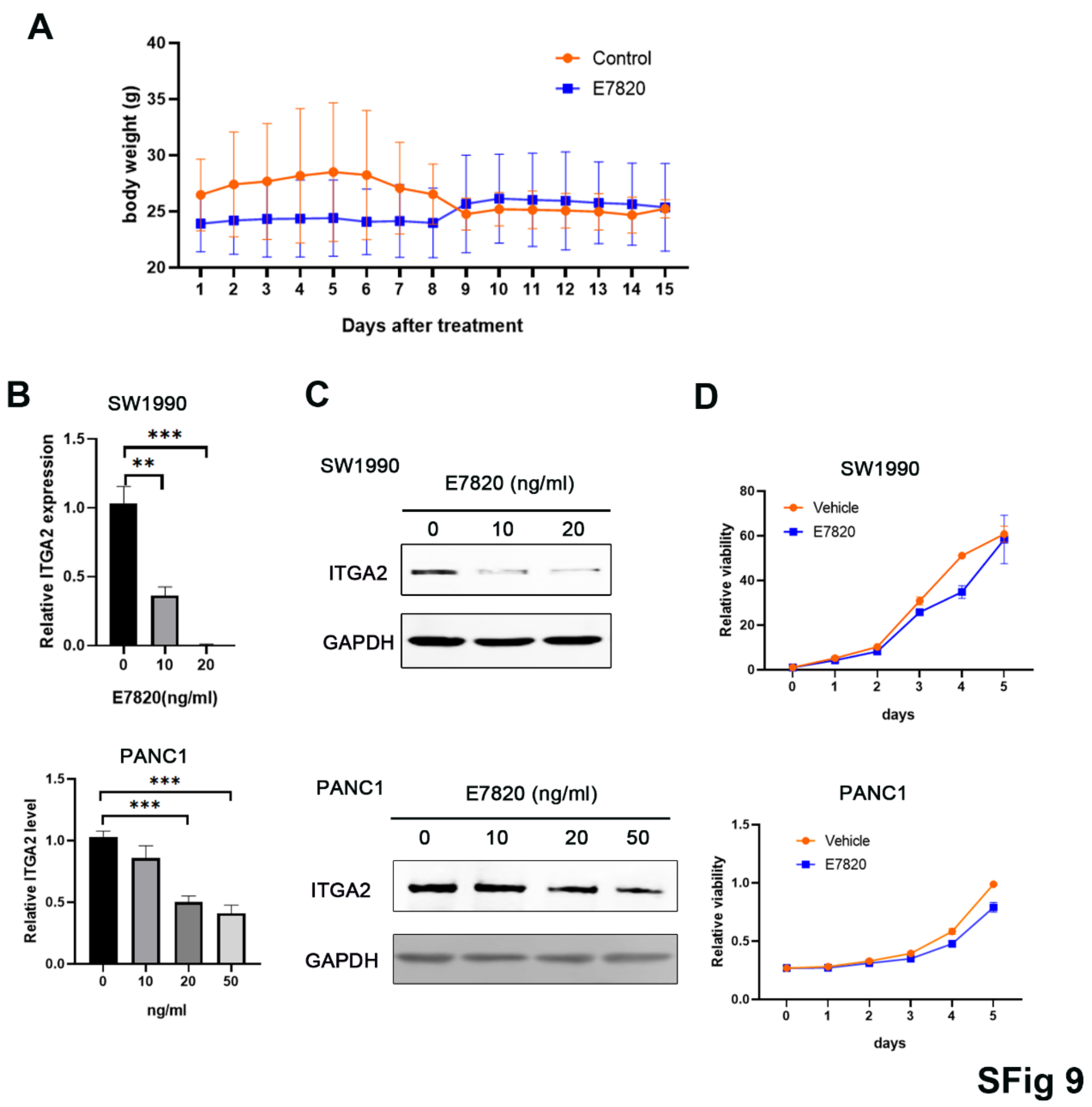

Supplementary Figure 9. E7820 decreases ITGA2 expression in PDAC cells

(A) Body weights of KC mice upon E7820 treatment for 1-15 days. Data shown are mean ± SD, n = 3. (B) Real-time PCR assay shows mRNA expression of ITGA2 in SW1990 and PANC1 cells treated with E7820, data shown are mean ± SD, n = 3, t test, **P<0.01, ***P<0.001. (C) Immunoblotting assay of E7820 treating SW1990 and PANC1 cells for ITGA2 protein expression. (D) The *in vitro* growth of SW1990 and PANC1 cells upon E7820 treatment was revealed by CCK8 assays.
