# Supplementary material for: Gene Coexpression Network Characterizing Microenvironmental Heterogeneity and Intercellular Communication in Pancreatic Ductal Adenocarcinoma: Implications of Prognostic Significance and Therapeutic Target
Source: Front Oncol. 2022 Jun 1;12:840474. doi: 10.3389/fonc.2022.840474 (PMC9198606; doi:10.3389/fonc.2022.840474)
Supplement: Supplementary file 2 [file Table_1.docx]

**Supplementary table**

**Table 1. Basic clinicopathologic data of tissue samples from patients with PDAC**

| **Parameter** | **No. of tissue samples** |
| --- | --- |
| Age |  |
| ＞60 | 30 |
| ≤60 | 36 |
| Sex |  |
| Male | 35 |
| Female | 31 |
| Tumor stage ^a^ |  |
| I A | 10 |
| I B | 24 |
| II A | 5 |
| II B | 15 |
| III | 1 |
| IV | 11 |
| Histological differentiation |  |
| Well | 8 |
| Moderate | 24 |
| Poor | 34 |

^a^ Tumor stage was classified according to the 7th edition of the American Joint Committee on Cancer (AJCC) Tumor Node Metastasis (TNM) classification of malignant tumors.
